# The phosphatidylinositol-3-phosphate 5-kinase inhibitor apilimod blocks filoviral entry and infection

**DOI:** 10.1371/journal.pntd.0005540

**Published:** 2017-04-12

**Authors:** Elizabeth A. Nelson, Julie Dyall, Thomas Hoenen, Alyson B. Barnes, Huanying Zhou, Janie Y. Liang, Julia Michelotti, William H. Dewey, Lisa Evans DeWald, Richard S. Bennett, Patrick J. Morris, Rajarshi Guha, Carleen Klumpp-Thomas, Crystal McKnight, Yu-Chi Chen, Xin Xu, Amy Wang, Emma Hughes, Scott Martin, Craig Thomas, Peter B. Jahrling, Lisa E. Hensley, Gene G. Olinger, Judith M. White

**Affiliations:** 1 Department of Cell Biology, University of Virginia, Charlottesville, Virginia, United States of America; 2 Integrated Research Facility, Division of Clinical Research, National Institute of Allergy and Infectious Diseases, National Institutes of Health, Frederick, Maryland, United States of America; 3 Laboratory of Virology, Division of Intramural Research, National Institutes of Health, Hamilton, Montana, United States of America; 4 Institute of Molecular Virology and Cell Biology, Friedrich-Loeffler-Institut, Greifswald–Insel Riems, Germany; 5 Division of Preclinical Innovation, National Center for Advancing Translational Sciences, National Institutes of Health, Bethesda, Maryland, United States of America; 6 Emerging Viral Pathogens Section, National Institute of Allergy and Infectious Diseases, National Institutes of Health, Frederick, Maryland, United States of America; George Mason University, UNITED STATES

## Abstract

Phosphatidylinositol-3-phosphate 5-kinase (PIKfyve) is a lipid kinase involved in endosome maturation that emerged from a haploid genetic screen as being required for Ebola virus (EBOV) infection. Here we analyzed the effects of apilimod, a PIKfyve inhibitor that was reported to be well tolerated in humans in phase 2 clinical trials, for its effects on entry and infection of EBOV and Marburg virus (MARV). We first found that apilimod blocks infections by EBOV and MARV in Huh 7, Vero E6 and primary human macrophage cells, with notable potency in the macrophages (IC_50_, 10 nM). We next observed that similar doses of apilimod block EBOV-glycoprotein-virus like particle (VLP) entry and transcription-replication competent VLP infection, suggesting that the primary mode of action of apilimod is as an entry inhibitor, preventing release of the viral genome into the cytoplasm to initiate replication. After providing evidence that the anti-EBOV action of apilimod is via PIKfyve, we showed that it blocks trafficking of EBOV VLPs to endolysosomes containing Niemann-Pick C1 (NPC1), the intracellular receptor for EBOV. Concurrently apilimod caused VLPs to accumulate in early endosome antigen 1-positive endosomes. We did not detect any effects of apilimod on bulk endosome acidification, on the activity of cathepsins B and L, or on cholesterol export from endolysosomes. Hence by antagonizing PIKfyve, apilimod appears to block EBOV trafficking to its site of fusion and entry into the cytoplasm. Given the drug’s observed anti-filoviral activity, relatively unexplored mechanism of entry inhibition, and reported tolerability in humans, we propose that apilimod be further explored as part of a therapeutic regimen to treat filoviral infections.

## Author summary

The recent outbreak of Ebola virus (EBOV) disease in Western Africa highlights the urgent need to develop therapeutics to help quell this devastating hemorrhagic fever virus, especially in resource-limited areas around the globe. Here we show that apilimod, an investigational drug that was well-tolerated in phase 2 clinical trials for rheumatoid arthritis, Crohn’s disease, and psoriasis, is a strong inhibitor of both EBOV and Marburgvirus infections in multiple cell types. Further work shows that apilimod blocks the entry of EBOV particles into the host cell cytoplasm and that it does so by blocking the particles from reaching their normal portal of entry, in Niemann-Pick C1-positive endolysosomes. Our findings are consistent with the identity of phosphatidylinositol-3-phosphate 5-kinase as the molecular target of apilimod, as the kinase and its product phosphatidylinositol 3,5-bisphosphate are required for the proper maturation of late endocytic organelles. Hence we propose that apilimod be further explored for repositioning as part of a therapeutic regimen to help ameliorate the sequelae of filoviral infections.

## Introduction

The epidemic of Ebola virus disease (EVD) that raged through Western Africa between 2013 and 2016 was the most severe filovirus disease epidemic in recorded history [1,2]. While several promising therapeutic antibodies [3–11] and novel small molecules [12–19] remain in development, no therapeutic is yet approved to treat patients with EVD. In the continuing pursuit of an anti- Ebola virus (EBOV) therapeutic, one strategy is to identify approved drugs that show anti-EBOV activity [20–28], with the goal of repurposing them for an anti-EBOV therapeutic, either alone or as part of a multi-component regimen [29–34].

Most of the approved drugs that have been identified as blocking EBOV infection inhibit the entry phase of the viral lifecycle [19–25,27,28]. Cell entry by EBOV is a complex process [35,36] entailing virus binding to cell surface attachment factors, internalization by macropinocytosis, processing by endosomal proteases, and transport to endolysosomes containing Niemann-Pick C1 (NPC1) [14,37], the intracellular receptor for EBOV [38]. Finally, EBOV fuses with the limiting membrane of NPC1^+^ endolysosomes [39–41], liberating its genome and associated proteins into the cytoplasm to begin replication.

The essential role of NPC1 in EBOV entry and infection was powerfully illuminated in a haploid genetic screen [37]. The same screen revealed other gene products critical for EBOV entry [42,43] including many involved in endosome and lysosome biogenesis and maturation. One of the latter proteins was phosphatidylinositol-3-phosphate 5-kinase (PIKfyve) [37], a lipid kinase that phosphorylates phosphatidylinositol-3-phosphate (PI3P) to generate phosphatidylinositol-3,5-bisphosphate (PI(3,5)P_2_). PIKfyve and PI(3,5)P_2_ are known to be critical for endosome maturation [44–53].

Apilimod is a small molecule that binds to and inhibits the phosphotransferase activity of PIKfyve [54]. The drug was developed as a suppressor of interleukin 12 and 23 production [55], and was tested in phase 2 clinical trials for treatment of Crohn’s disease [56,57], psoriasis [58], and rheumatoid arthritis [59]. Although no clinical benefit has yet been reported, apilimod is deemed to be well tolerated in humans. We chose to test whether apilimod could inhibit infections by EBOV and Marburg virus (MARV) for three reasons. The first was that apilimod binds [54] to the EBOV entry factor PIKfyve [37]. The second was because apilimod emerged from a blinded screen of 35 drugs (S1 Fig; S1 Table), which were selected as potential inhibitors based upon hypotheses of drugable targets and from theoretical considerations of pathways possibly involved in the EBOV life cycle. The third reason was because apilimod is well tolerated in humans. We find that apilimod inhibits infection by both EBOV and MARV, being notably effective in primary human macrophages, which are initial targets of filoviral infection [60,61]. Mechanistic studies revealed that apilimod blocks EBOV entry into the cell cytoplasm by working through PIKfyve and that its effect is to block viral particle trafficking to NPC1^+^ endolysosomes, the site of EBOV fusion [39–41]. Hence we propose that apilimod be further explored as part of a cocktail of small molecules to combat EVD.

## Materials and methods

### Cells

Vero E6 (African green monkey kidney; ATCC 1586) cells were obtained from the American Type Culture Collection (Manassas, VA). Huh 7 (human hepatocellular carcinoma) cells were obtained from Dr. Hideki Ebihara (National Institute of Allergy and Infectious Diseases (NIAID), Rocky Mountain Laboratories, Hamilton, MT). Peripheral blood mononuclear cells (PBMCs) were prepared from human whole blood (Biological Specialty Corporation; Colmar, PA; Cat # 3100-03-04) and human monocyte-derived macrophages (hMDM) were generated from peripheral blood mononuclear cells at the Integrated Research Facility (IRF) immunology core laboratories as described previously [62,63]. hMDM were characterized by flow cytometric analysis for expression of major macrophage markers, including human leukocyte antigen-D related, CD11b, CD14, CD163, and CD86, to confirm that the hMDM population was mature and highly purified [63]. HEK 293T/17 (Human embryonic kidney; ATCC CRL-11268 via University of Virginia Tissue Culture Facility) and BSC-1 (Grivet monkey kidney; gift from Dr. Xiaowei Zhuang, Harvard University, Cambridge, MA) cells were maintained in growth medium: high glucose Dulbecco's Modified Eagle Medium (DMEM) supplemented with 1% L-glutamine, 1% sodium pyruvate, and 1% antibiotic/antimycotic, all from Gibco Life Technologies (Carlsbad, CA), and either 10% supplemented calf serum (SCS; Hyclone, GE Healthcare Bio-Sciences, Pittsburgh, PA) for HEK 293T/17 cells or 10% fetal bovine serum (FBS, Seradigm, VWR International, Radnor, PA) or 10% cosmic calf serum (CCS, Hyclone) for BSC-1 cells.

### Drugs

Toremifene citrate (CAS 89778-27-8) was purchased from Sigma-Aldrich (St. Louis, MO; Cat# T7204-25MG) and apilimod (CAS 541550-19-0) was purchased from Axon MedChem (Groningen, NL; Cat# 1369). Nocodazole (CAS 31430-18-9) was purchased from Sigma-Aldrich (St. Louis, MO; Cat # M1404-2MG).

### Virus isolation

All procedures using infectious EBOV/Mak or MARV were performed under biosafety level 4 (BSL-4) conditions at the IRF. The C05 isolate of the Makona variant of EBOV (EBOV/Mak; full designation: Ebola virus/H.sapiens-tc/GIN/2014/Makona-C05; GenBank: KX000398) and Marburg Angola virus (MARV; full name: Marburg virus/H.sapiens-tc/AGO/2005/Ang-1379v; GenBank: N/A) were propagated in BEI NR-596 Vero E6 cells and used after one or two passages.

### Cell-based efficacy and cytotoxicity testing of EBOV antiviral agents

The drug screen method was performed as described previously [63]. Briefly, Vero E6 and Huh 7 cells were seeded in 96-well plates at a density of 3 x 10^4^ cells/well, and hMDMs were plated at a density of 1 x 10^5^ cells/well 24 h prior to the addition of drugs. For each cell type, cells were plated in 1 black opaque 96-well plate, for the evaluation of drug cytotoxicity, and 2 clear bottom, 96-well Operetta plates, for the evaluation of drug efficacy. Drugs dissolved in dimethyl sulfoxide (DMSO; Sigma-Aldrich, St. Louis, MO) were diluted in DMEM with 10% FBS with the final DMSO concentration not exceeding 0.05%. The drug solutions were diluted two-fold in an 8-point dilution series and transferred to cell plates 1 h prior to virus infection. Efficacy plates for each cell type were infected with EBOV or MARV at a multiplicity of infection (MOI) of 0.5. After 48 h, cells were fixed with 10% neutral-buffered formalin.

Chemiluminescent enzyme-linked immunosorbent assay was used to determine virus activity. EBOV was detected with a mouse antibody against the EBOV VP40 matrix protein (B-MD04-BD07-AE11, made by US Army Medical Research Institute of Infectious Diseases, Frederick MD under Centers for Disease Control and Prevention contract) [3] and MARV was detected with a mouse antibody against the MARV VP40 protein (Cat# IBT 0203–012, IBT Bioservices, Rockville, MD) for 1–2 h at 37°C. Cells were stained with a secondary antibody, anti-**mouse** IgG, peroxidase labeled antibody (Cat# 074–1802, KPL Inc., Gaithersburg, MD). Luminescence was detected using Pico chemiluminescent Substrate (Thermo Fisher Scientific Inc., Rockford, IL) and an Infinite M1000 Pro plate reader (Tecan, Morrisville NC).

For quantitation of drug toxicity, 1 black opaque cell plate for each cell type was mock infected (no virus) and treated with drug dilutions under the same conditions as the infected cells. After 48 h, cell viability was measured using the CellTiter Glo Luminescent Cell Viability Assay kit according to the manufacturer’s protocol (Promega, Madison, WI). Luminescence was read on an Infinite M1000 Pro plate reader.

Following background subtraction, inhibition was measured as percent relative to untreated infected cells. Non-linear regression analysis was performed, and IC_50s_ were calculated from fitted curves (log [agonist] vs response [variable slope] with constraint to remain above 0; GraphPad Software, La Jolla, CA). Error bars of dose-response curves represent the standard deviation of three replicates.

### Preparation and assay of EBOV entry reporter VLPs

Entry reporter viral-like particles (VLPs) bearing GP from the Yambuku-Mayinga isolate of EBOV were prepared as described previously [24,25,41]. In brief, HEK 293T/17 cells (~80% confluent) were transfected with cDNAs encoding EBOV GP, VP40, mCherry-VP40, and β-lactamase-VP40 (βlam-VP40). The cell medium was collected 24 and 48 h post-transfection and cleared of debris. VLPs in the cleared medium were then pelleted through a 20% sucrose cushion by centrifugation, resuspended in HM buffer (20 mM HEPES, 20 mM MES, 130 mM NaCl, pH 7.4), and repelleted. The final VLP pellet was resuspended (1:100 starting volume of medium) in 10% sucrose-HM. The total protein concentration of the VLPs was determined by bicinchoninic acid (BCA) assay. All entry-reporter VLP preparations were assessed by western blot analyses (for the presence of GP as well as EBOV VP40) and titered on HEK 293T/17 cells to confirm entry competency.

The VLP entry assay scores the ability of βlam-VP40 (from incoming entry reporter VLPs) to cleave a βlam substrate preloaded into the target cell cytoplasm; this only occurs if the VLP fuses with an endosome. The assay was performed as described previously [24,25,41]. In brief, 30,000 HEK 293T/17 cells or BSC-1 cells were seeded per well in a clear 96-well plate. 18–24 h post seeding, the cells (~80%–90% confluent) were treated with the indicated concentration of apilimod (Axon MedChem; DMSO for mock) diluted in Opti-MEM I (OMEM, Gibco Life Technologies, Thermo Fisher Scientific) for 1 h at 37°C in a 5% CO_2_ incubator. VLPs diluted in OMEM (with DMSO or the same concentration of apilimod) were bound to the cells by spinfection (250× g) for 1 h at 4°C. After 3 h in a 37°C, 5% CO_2_ incubator, the βlam substrate CCF2-AM (Life Technologies, via ThermoFisher Scientific, Waltham, MA, USA) was loaded into the cells using 20 or 5 mM Probenecid (MP Biomedicals via ThermoFisher Scientific, Waltham, MA, USA), for BSC-1 or HEK 293T/17 cells, respectively. The cells were incubated overnight at RT and then fixed and analyzed by flow cytometry.

To measure corresponding cell viability, 3 x 10^4^ HEK 293T/17 cells, seeded and grown as above but in 96-well opaque white plates were treated as above for VLP entry, but without addition of VLPs or CCF2-AM. Following overnight incubation at RT (as above), the medium was removed and replaced with 50 μL of fresh medium per well. Fifty microliters (per well) of CellTiter-Glo 2.0 (Promega, Madison WI, USA) was then added. After shaking for 2 min at RT at 575 rpm on a Jitterbug orbital shaker (Boekel Scientific, via ThermoFisher Scientific, Waltham, MA, USA), the plate was incubated at RT for 10 min, after which the luminescent signal was detected using a BioTek Synergy HT plate reader (BioTek, Winooski, VT, USA).

### Preparation and assay of transcription/replication competent VLPs

Transcription/replication-competent virus-like particles (trVLPs) were prepared as described [25,64,65]. In brief, HEK 293T/17 cells were seeded in six well plates and transfected 24 h later (when ~50% confluent) using TransIT-LT1 (Mirus, Madison, WI, USA) with pCAGGS-NP, pCAGGS-VP35, pCAGGS-VP30, pCAGGS-L, a tetracistronic minigenome plasmid, and pCAGGS-T7 polymerase. The minigenome plasmid encodes *Renilla* luciferase, as well as the matrix protein VP40, the nucleocapsid associated protein VP24, and the GP from EBOV. 24 h post transfection, the medium in each well was replaced with 4 mL fresh growth medium containing 5% FBS. 96 h after transfection, the medium (containing trVLPs harboring the *Renilla* luciferase-containing minigenome) was harvested, pooled, and cleared of cellular debris by centrifugation for 5 min at 800× g and used for trVLP assays as described below.

The trVLP assay measures the ability of trVLPs containing a Renilla luciferase-encoding tetracistronic EBOV minigenome to infect target cells pretransfected with plasmids encoding proteins to enhance trVLP entry (the adhesion factor Tim-1) and (other plasmids) to support replication of the minigenome. If trVLPs enter target cells, the minigenome is replicated and transcribed, leading to *Renilla* luciferase reporter activity [64,65]. In brief: Cells were pretreated with apilimod (Axon MedChem; DMSO for mock) as described above. The pretreatment solution was then removed and replaced with 100 μL trVLPs diluted to 200 μL in growth medium containing 10% SCS and the indicated concentration(s) of apilimod (DMSO for mock). The cells were then incubated for 48 h at 37°C in a 5% CO_2_ incubator, after which the medium was replaced with 40 μL of fresh growth medium containing 10% SCS. 40 μL of RenillaGlo substrate (Promega, Madison, WI, USA) was then added to each well and the plate immediately analyzed on a GloMax plate reader (Promega, Madison, WI, USA).

To assess cell viability in corresponding samples without trVLPs, the pretreatment solution was removed and replaced with 200 μL fresh growth medium containing 10% SCS and the indicated concentrations of apilimod (DMSO for mock). The cells were then incubated for 48 h at 37°C in a 5% CO_2_ incubator, after which the medium was replaced with 40 μL of fresh growth medium containing 10% SCS. 40μL of CellTiter-Glo 2.0 (Promega) was then added to each well and the plate placed on a Jitterbug orbital shaker (575 rpm) for 2 min at RT. The plate was then incubated at RT for 10 min, after which the luminescent signal was detected using a Synergy HT (BioTek, Winooski, VT, USA) plate reader.

### Assay of endosomal pH

BSC-1 cells were seeded in 35mm glass bottom dishes (MatTek, Ashland, MA) that were coated with 20 μg/mL fibronectin (Sigma-Aldrich, St. Louis, MO, USA). The next day, when the cells were 90–100% confluent, the cells were treated with the indicated drug at the indicated concentration, diluted in growth medium containing 10% cosmic calf serum, for 3 h at 37°C in a 5% CO_2_ incubator. Acridine Orange (Life Technologies, Thermofisher Scientific, Waltham, MA, USA) was added directly to each dish to reach a final concentration of 6.6 **μg**/mL. The cells were incubated at 37°C in a 5% CO_2_ incubator for 20 min and then were washed 3 times with phosphate buffered saline (PBS), 5 min per wash. Cell imaging medium [Live cell imaging solution (Molecular Probes, Cat# A14291DJ, Thermo Fisher Scientific, Waltham, MA) containing 10% FBS and 4.5 g/L glucose] was added to the dishes and images were taken using a Nikon C1 laser scanning confocal unit attached to a Nikon Eclipse TE2000-E microscope with a 100X, 1.45-numerical-aperature (NA) Plan Apochromat objective (Nikon, Melville, NY).

### Assay of cholesterol accumulation

BSC-1 cells were seeded in 35mm glass bottom dishes (MatTek, Ashland, MD, USA) that had been coated with 20 μg/mL fibronectin (Sigma-Aldrich). The next day, when the cells were 90–100% confluent, the cells were treated with the indicated drug at the indicated concentration plus 0.05 μM TopFluor Cholesterol (Avanti Polar Lipids, Alabaster, AL), diluted in serum-free growth medium, for 18 h at 37°C in a 5% CO_2_ incubator. Following incubation, the cells were gently rinsed once with PBS and cell imaging medium (Live cell imaging solution (Molecular Probes) containing 10% FBS and 4.5 g/L glucose) was added to the dishes. The cells were incubated at 37°C in a 5% CO_2_ incubator for 30 min. Images were then taken using a 60X /1.45 numerical aperture (NA) Nikon Plan Apo total internal reflection fluorescence oil immersion objective attached to a Nikon Eclipse TE2000-E microscope equipped with a Yokogawa CSU 10 spinning-disk confocal unit, a 512-by-512 Hamamatsu 9100c-13 EM-BT camera, a motorized stage maintained at 37°C, and a Nikon Perfect Focus system.

### Assays of VLP trafficking

VLP trafficking experiments were performed in BSC-1 cells essentially as described previously [41] with the following minor modifications. Cells were pretreated with apilimod or nocodazole (indicated concentrations) diluted in OMEM for 1h at 37°C prior to VLP addition. VLPs at 0.5 μg/well were bound to the cells by spinfection (250 x g) for 1 h at 4°C. After incubation at 37°C (CO_2_ incubator) for the indicated times, the cells were fixed and washed. Next, primary antibodies (1:1000 rabbit α-NPC1, (Abcam) or 1:1000 mouse α-early endosome antigen 1 (EEA1), BD Biosciences, San Jose, CA) were added for 45 min at room temperature (RT) and, following washing, secondary antibodies (1:1500 α-mouse or α-rabbit AlexaFluor 488, Life Technologies, Thermo Fisher Scientific) were added for 30 min at RT. The cells were washed and the coverslips were mounted overnight on glass slides using ProLong Gold Antifade reagent (Life Technologies, Thermo Fisher Scientific). The coverslips were then sealed and images were taken using a Nikon C1 laser scanning confocal unit attached to a Nikon Eclipse TE2000-E microscope with a 100X, 1.45-NA Plan Apochromat objective. Colocalization of VLPs (red, mCherry-VP40) and endosomal markers (green, NPC1 or EEA1) was assessed as Manders coefficients. Statistics were analyzed using GraphPad Prism 7. Normality of the data was assessed using the D’Agostino & Pearson normality test. Significance of normally distributed data was determined by T-test, and significance of non-normally distributed data was determined by Mann-Whitney test.

### Assay of cathepsin B+L activity

Cathepsin B+L activity was assayed as described previously [20,21,24]. (2S,3S)-*trans*-epoxysuccinyl-L-leucylamido-3-methylbutane ethyl ester (EST, Cat # 330005, Calbiochem, EMD Millipore, Billerica, MA), an inhibitor of cathepsin B,H, and L, was used as a positive control for inhibition at the indicated concentration. Data are displayed as fluorescence units (Ex 360/Em 460).

### Screening of drugs using rgEBOV-luc2

Thirty-five drugs obtained from the National Center for Advancing Translational Sciences (NCATS) were dissolved in DMSO at 500 μM. Drugs were diluted in DMEM (Life Technologies, Thermo Fisher Scientific) supplemented with 2 mM L-Glutamine (Q; Life Technologies, Thermo Fisher Scientific) and 100 U/ml penicillin and 100 μg/ml streptomycin (PS; Life Technologies, Thermo Fisher Scientific). Drugs were added to confluent Vero E6 cells. Drugs and cells were then incubated at 37°C and 5% CO_2_ in a humidified incubator in 96-well plates for final concentrations of 10, 1, or 0.1 μM in a final volume of 100 μl DMEM/PS/Q with 2% FBS (Life Technologies, Thermo Fisher Scientific). Cells were returned to the incubator for 2 h.

For efficacy studies, 50 μl DMEM/PS/Q containing 1x10^3^ TCID_50_ of recombinant EBOV expressing firefly luciferase from an additional transcriptional unit (rgEBOV-luc2, Genbank Accession number KF990214.1) [66] was added to the cells. At 48 h post-inoculation the supernatant was removed and 100 μl GloLysis buffer (Promega) was added to the cells and incubated for 10 min at RT. Afterwards, 40 μl lysate was added to 40 μl BrightGlo reagent (Promega) in white opaque 96 well plates, and reporter activity was measured using a GloMax luminometer.

For cytotoxicity studies, 50 μl of DMEM/PS/Q without virus was added to the cells following the 2 h pre-incubation with drugs, and cells were returned to the incubator. At 48 h, 100 μl of supernatant was removed, and 50 μl of CellTiterGlo reagent (Promega) was added to the cells. Cells were incubated for 2 min on an orbital shaker at 60 RPM, and then for an additional 10 min without shaking at RT. Supernatants were transferred to white opaque 96-well plates, and reporter activity was measured using a GloMax luminometer (Promega). Ribavirin at final concentrations of 1 mg/ml, 100 μg/ml, and 10 μg/ml, as well as DMSO at concentrations corresponding to the DMSO concentrations found in the drug dilutions served as controls. All experiments involving infectious rgEBOV-luc2 were performed in the maximum containment laboratory of the Rocky Mountain Laboratories, National Institutes of Health, Hamilton, MT, following approved protocols.

### Production of MLV-luciferase (luc) pseudovirus particles

HEK 293T/17 cells were seeded at a density of 3 x 10^6^ cells per 10 cm plate. The next day, when the cells were approximately 60% confluent, the media above the cells was replaced with 6mL OMEM and the cells were transfected with 2.4 μg pTG-luc, 1.2 μg pCMV-MLVgag-pol, 1.2 μg pGPΔmucin (encoding Ebola GP deleted for its mucin domain), and 1.2 μg of MLV-gag-βlam diluted to 300 μL in OMEM (per plate), using 18 μL Lipofectamine 2000 (Invitrogen, ThermoFisher Scientific, Walthan, MA) diluted to 300 μL in OMEM (per plate). 4h post transfection, 6 mL of antibiotic-free growth medium containing 10% SCS was added to each plate, and the cells were incubated for 48 h at 37°C in a CO_2_ incubator. Cell medium containing pseudovirus was then collected, pooled, and cleared of cellular debris by centrifugation at 250 x g for 7 min. The clarified supernatant containing pseudovirus was then passed through an 0.45 μm filter and the pseudoviruses were concentrated 100-fold by high-speed centrifugation through a 25% sucrose cushion in HM buffer (20mM HEPES, 20mM MES, 130mM NaCl, pH7.4) for 75 min at 103,745 x g. The final pseudovirus pellet was resuspended in growth medium (100-fold concentrated from harvest supernatant).

### Over-expression of GFP-PIKfyve and MLV-Luc infection assay

HEK 293T/17 cells were seeded at a density of 5 x 10^5^ cells per well in 6-well plates. When the cells were ~50% confluent (~18–24 h post seeding), they were transfected with plasmids encoding GFP-PIKfyve or pEGFP-Cl using TransIT LT1 transfection reagent (Mirus, Madison, WI) following the manufacturer’s instructions. 18 h post transfection, the cells were re-seeded in 96 well opaque white plates (BD Falcon, ThermoFisher Scientific, Waltham, MA) at a density of 3 x 10^4^ cells per well. Transfection was confirmed by fluorescence microscopy. 18 h post re-seeding, the cells were pretreated for 1 h at 37°C with apilimod. MLV-luciferase particles pseudotyped with EBOV GPΔmucin were added to the cells in the presence of apilimod, and infection was allowed to proceed for 48 h at 37°C. The cells were then washed once with PBS and overlaid with 50μL PBS. Luciferase activity was then immediately assayed by adding 50μL of Britelite plus (Perkin Elmer, Waltham, MA) and reading on a Glomax plate reader (Promega, Madison, WI, USA) following the manufacturer’s instructions.

## Results

### Apilimod blocks EBOV and MARV infection of multiple cell types

**Fig 1:**
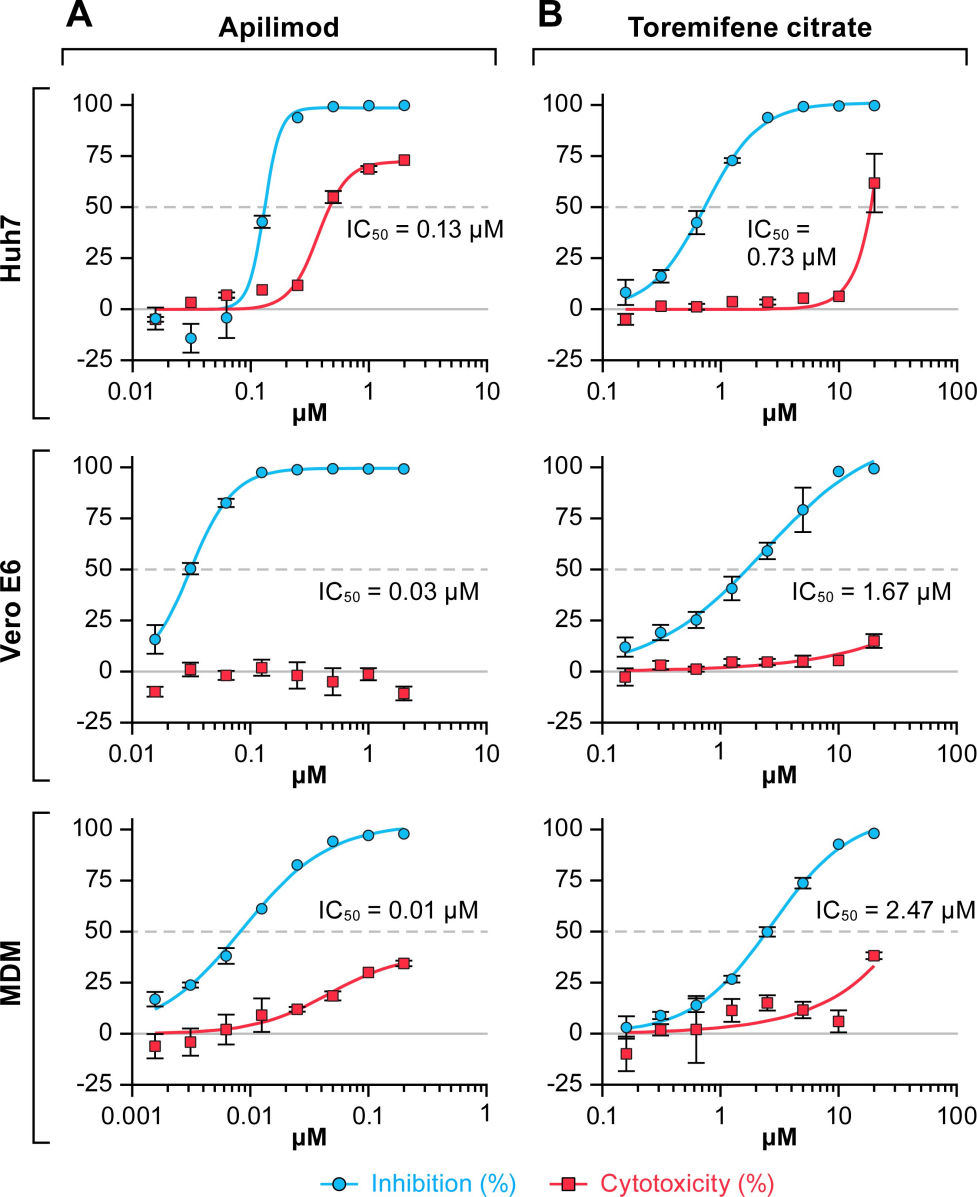
Apilimod blocks EBOV infection of Huh 7 and Vero E6 and human primary macrophages. Huh 7 and Vero E6 cells and human monocyte-derived macrophages (hMDM) were treated for 1 h with apilimod (A) or toremifene citrate (B). Two-fold dilutions of the drugs were tested in an 8-point dose-response curve. Then cells were infected at a multiplicity of infection (MOI) of 0.5 for 48 h. Antiviral activity is shown in blue and cytotoxicity is shown in red. The experiment was run on duplicate plates with triplicate wells per dose (mean ± SD; n = 3). The experiment was repeated on 2–4 days. A single representative graph is shown.

**Fig 2:**
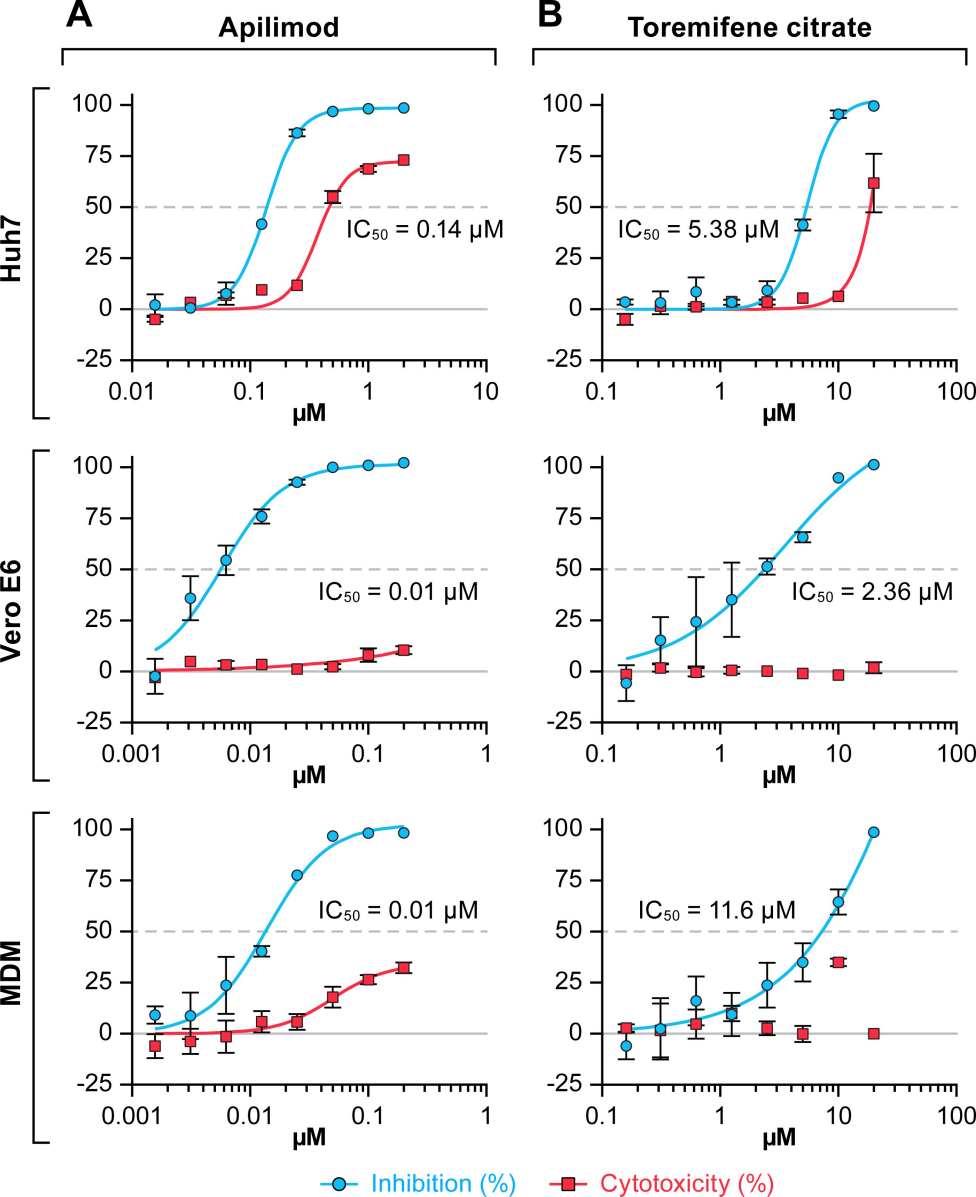
Apilimod blocks MARV infection of Huh 7 and Vero E6 cells and human primary macrophages. Huh 7 and Vero E6 cells and human monocyte-derived macrophages (hMDM) were treated for 1 h with apilimod (A) or toremifene citrate (B). Two-fold dilutions of the drugs were tested in an 8-point dose-response curve. Then cells were infected at a multiplicity of infection (MOI) of 0.5 for 48 h. Antiviral activity is shown in blue and cytotoxicity is shown in red. The experiment was run on duplicate plates with triplicate wells per dose (mean ± SD; n = 3). The experiment was repeated on 2–4 days. A single representative graph is shown.

We first tested whether apilimod blocks EBOV infection in cell cultures. Apilimod blocked EBOV infection of Huh 7 (liver) cells, Vero E6 (kidney) cells, and primary human monocyte-derived macrophages (hMDMs) (Fig 1) with 6- to 247-fold higher activity than the positive control, toremifene citrate [20,22]. Apilimod also blocked MARV infection of the same cell types (Fig 2) with 38- to 1160-fold higher activity than the positive control. Apilimod was notably potent (IC_50_, 10 nM) against both filoviruses in hMDMs (Figs 1 and 2, Table 1). While similar potency (IC_50_, 15–25 nM) was seen in Vero E6 cells, apilimod was ~10-fold less potent (IC_50_, 140 nM) in Huh 7 cells (Fig 1, Fig 2 and Table 1).

**Table 1:** Effects of Apilimod on Ebola Virus and Marburg Virus Replication

Virus	Cell type	CC_50_ (μM)^a^	IC_50_ (μM)^a^	SI^b^
EBOV	Huh 7	0.48 ± 0.08	0.136 ± 0.040	3.5
EBOV	Vero E6	> 2.00	0.025 ± 0.010	> 80.0
EBOV	hMDM	> 2.00	0.009 ± 0.001	> 222.0
MARV	Huh 7	0.50 ± 0.1	0.140 ± 0.050	3.6
MARV	Vero E6	> 2.00	0.015 ± 0.003	> 133.0
MARV	hMDM	> 2.00	0.010 ± 0.004	> 200.0

^a^CC_50_ and IC_50_ values are mean values ± standard deviation from 4 to 8 dose response curves.

^b^SI = CC_50_/IC_50_; Abbreviations: CC_50_, concentration with 50% cell viability; EBOV, Ebola virus; IC_50_, 50% inhibitory concentration; MARV, Marburg virus; hMDM, human primary monocyte-derived macrophage; MOI, multiplicity of infection; SI, selectivity index

### Apilimod blocks EBOV particle entry into the host cell cytoplasm

**Fig 3:**
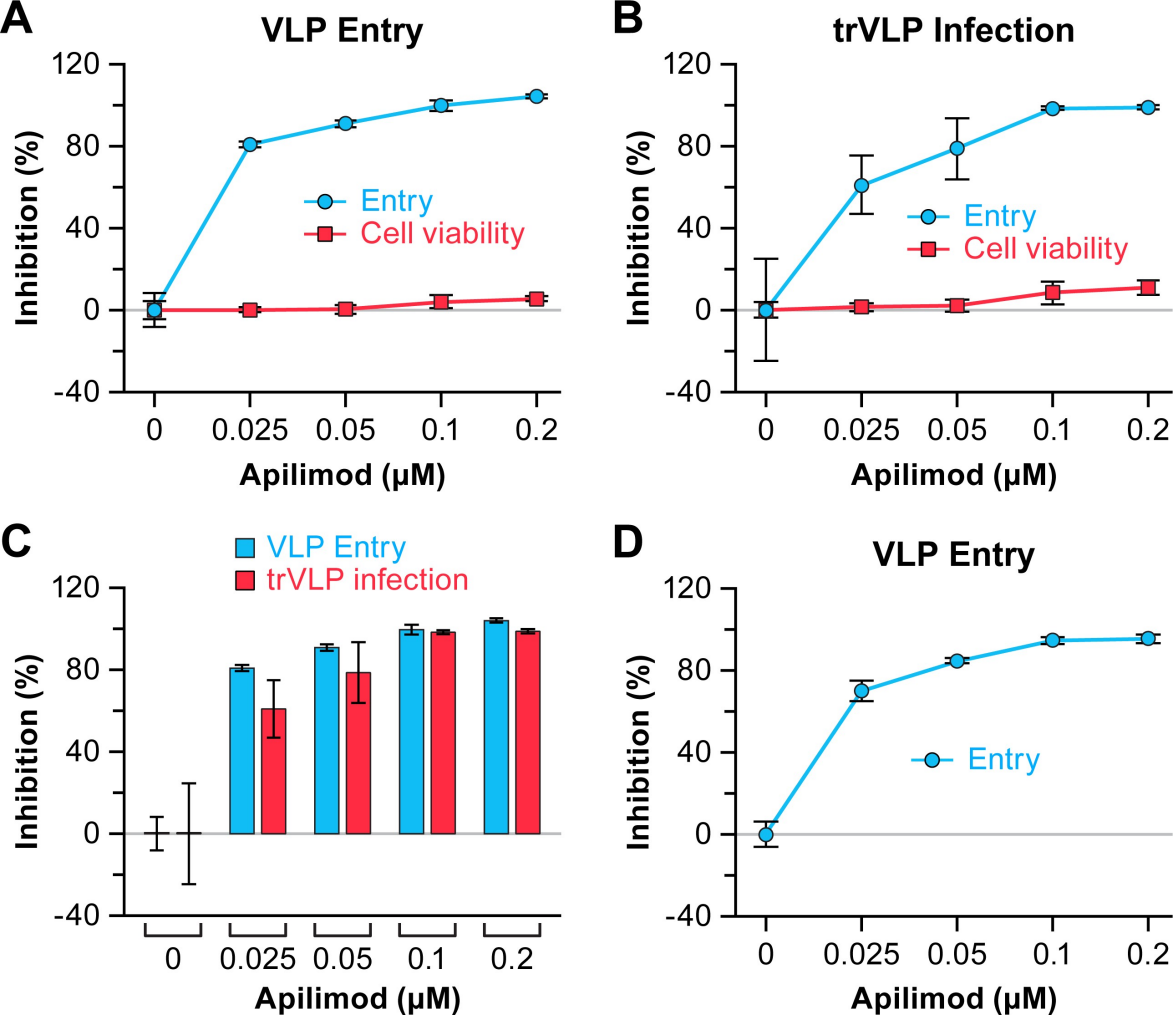
Apilimod inhibits VLP entry and trVLP infection with similar potency. (A) HEK 293T/17 cells were pretreated with the indicated concentration of apilimod or DMSO (0 μM) for 1 h at 37°C. VLPs were bound to the cells by spinfection in the presence of the indicated concentration of apilimod or DMSO. After incubation at 37°C (3 h), VLP entry was assayed. To assess cell viability, parallel 293T/17 cells were treated as for entry, but without VLPs or CCF2 loading. After 3 h at 37°C, cell viability was determined. (B) Cells (HEK 293T/17) pretreated as in (A) were infected with trVLPs (in the presence of the indicated concentration of apilimod) for 48 h at 37°C and infection by trVLPs was then assayed. To measure cell viability, parallel HEK 293T/17 cells were pretreated as above and then mock infected (± the indicated concentration of apilimod) for 48 h at 37°C followed by determination of cell viability. Antiviral activity is shown in blue and cytotoxicity is shown in red. (C) Comparison of normalized VLP entry (blue bars) and trVLP infection (red bars) inhibition; data are from (A) and (B). Data are averages of triplicate samples ± SD. Similar results for parallel tests of VLP entry and trVLP inhibition were observed in two additional experiments. Cell viability was tested in one of these experiments, and similar results were obtained. (D) BSC-1 cells were treated with apilimod and VLP entry was assayed as in (A). Data are averages of triplicate samples ± SD. Similar results were observed in an additional experiment.

To begin to probe the mechanism by which apilimod blocks EBOV infection, we directly compared dose-response profiles for blocking EBOV entry and replication using entry reporter VLPs [24] and trVLPs [64], respectively. Both sets of VLPs bore the GP from the Mayinga isolate of EBOV. Apilimod blocked EBOV particle entry (Fig 3A) and replication (Fig 3B) with similar dose-response profiles (Fig 3C). This finding suggested that apilimod blocks the entry phase of the filoviral lifecycle.

### Inhibition of EBOV entry by apilimod is mediated by PIKfyve

**Fig 4:**
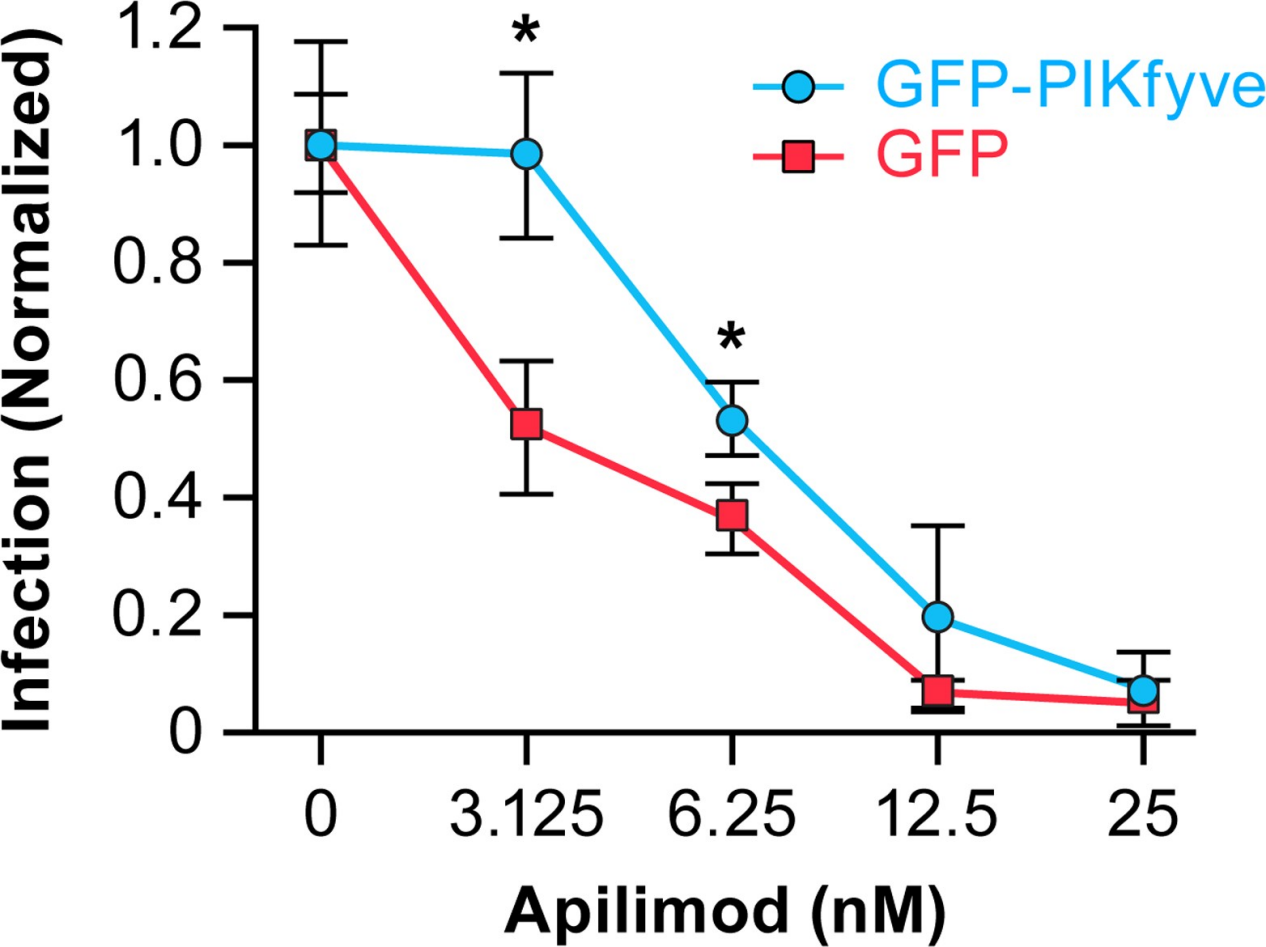
Apilimod inhibits EBOV GP-mediated entry in a PIKfyve-dependent manner. HEK 293T/17 cells in 6-well plates were transiently transfected with plasmids encoding GFP-PIKfyve or (as control) GFP. After 18 hr, the cells were reseeded into 96-well plates. After a further 18 hr in culture, the cells were pretreated with the indicated concentration of apilimod for 1 hr at 37°C. MLV-luc-GPΔmucin pseudovirions were then added to the cells in the presence of the indicated concentration of apilimod (or DMSO) and the cells incubated for 48 hr at 37°C, at which point infection was assayed as described in the Methods section. Data indicate averages ± SD of triplicate samples; * indicates p < 0.05. Similar results were obtained in a repeat experiment.

Since apilimod targets PIKfyve [54], since PIKfyve is required for EBOV entry and infection [37], and since apilimod blocks EBOV entry and infection (Figs 1–3), we reasoned that apilimod blocks EBOV entry and infection by targeting PIKfyve. To test this hypothesis we over-expressed PIKfyve (GFP-PIKfyve) and compared the dose response needed for apilimod to block EBOV-GP mediated pseudovirus infection. As predicted, and as seen in Fig 4, higher doses of apilimod were needed to achieve similar levels of inhibition of EBOV GP-mediated infection in GFP-PIKfyve vs. GFP expressing cells. This supports our proposal that apilimod blocks EBOV entry and infection through a PIKfyve-dependent pathway.

### Apilimod blocks EBOV particle trafficking to the site of fusion

**Fig 5:**
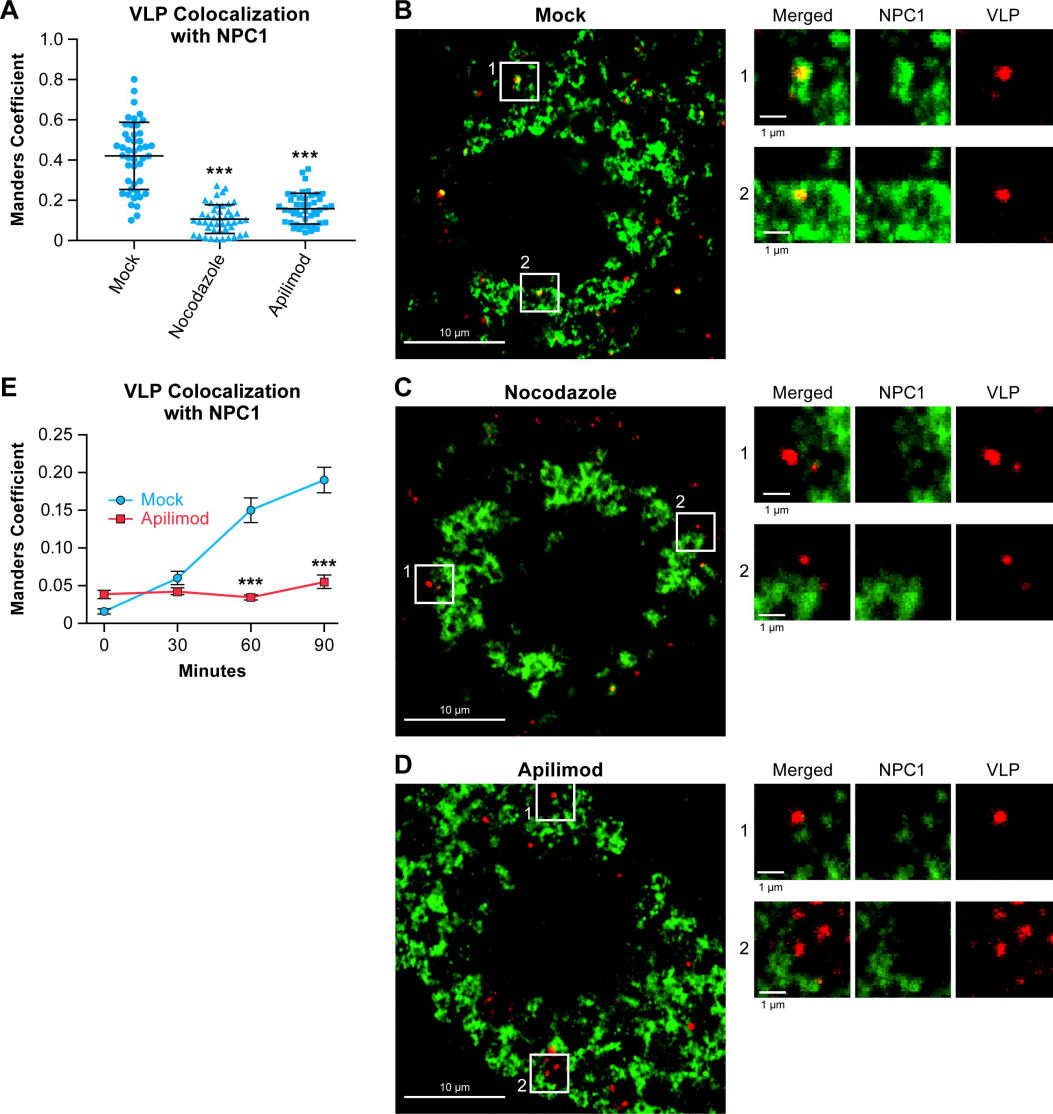
Apilimod inhibits trafficking of EBOV-GP VLPs to NPC1^+^ endolysosomes. (A-D) BSC-1 cells were pretreated with DMSO (Mock), 20 μM nocodazole, or 0.2 μM apilimod for 1 h at 37°C. VLPs were then bound to cells by spinfection in the presence of the indicated drug, and the cells were then washed and incubated in the presence of the indicated drug for 90 min at 37°C. The cells were then washed, fixed, permeabilized, stained, and analyzed for VLP colocalization with NPC1. (A) Average Manders colocalization coefficients (± SD) from 2 experiments (n = 45 fields each treatment). Each data point (blue dot, triangle or square) represents the Manders colocalization coefficient for 1 image field. (B-D) Representative micrographs of cells treated with (B) DMSO, (C) 20 μM nocodazole, or (D) 0.2 μM apilimod. Scale bars, 10 μm (primary images) and 1μm (insets). ***p<0.001. (E) VLP colocalization with NPC1 was monitored as above except that samples were fixed at 0, 30, 60 or 90 min. Data are the Manders colocalization coefficients from 24–29 microscope fields per sample. Values are averages ± SEM. Apilimod-treated samples were statistically different from mock (***p < 0.0001) at 60 and 90 min.

Recent work has shown that EBOV traffics deep in the endocytic pathway, to NPC1^+^ endolysosomes, for fusion and entry [39–41]. Therefore we asked whether apilimod prevents EBOV VLPs from reaching NPC1^+^ endolysosomes. We used BSC-1 cells for these experiments as they are more suitable (flatter and more adherent) for immunofluorescence analysis than the HEK 293T/17 cells used in previous experiments (Figs 3 and 4). We first demonstrated that apilimod blocks EBOV VLP entry into BSC-1 cells with the same approximate dose-dependency as its effects in HEK 293T/17 cells (Fig 3D). Given that, we next asked if apilimod blocks trafficking of EBOV GP VLPs to NPC1^+^ endolysosomes in BSC-1 cells. As seen in Fig 5, this was, indeed, the case. Apilimod blocked EBOV VLP trafficking to NPC1^+^ endolysosomes to a similar extent as nocodazole, a microtubule destabilizer that is known to block traffic between early and late endosomes [67].

The findings presented in Fig 5A–5D were obtained after allowing VLPs pre-bound to the cell surface to enter cells for 90 min at 37°C. This time point was chosen based on our extensive prior analysis of the time courses of EBOV VLP co-localization with NPC1^+^ endolysosomes and entry into BSC-1 cells [41]. To assure that apilimod did not accelerate VLP trafficking to NPC1^+^ endolysosomes, we analyzed co-localization of VLPs at various times up to 90 min in cells treated or not treated with apilimod. As seen in Fig 5E, at no point during this time course were VLPs seen to associate with NPC1^+^ endolysosomes in apilimod-treated cells, supporting our conclusion that apilimod blocks trafficking of EBOV particles to NPC1^+^ endolysosomes.

**Fig 6:**
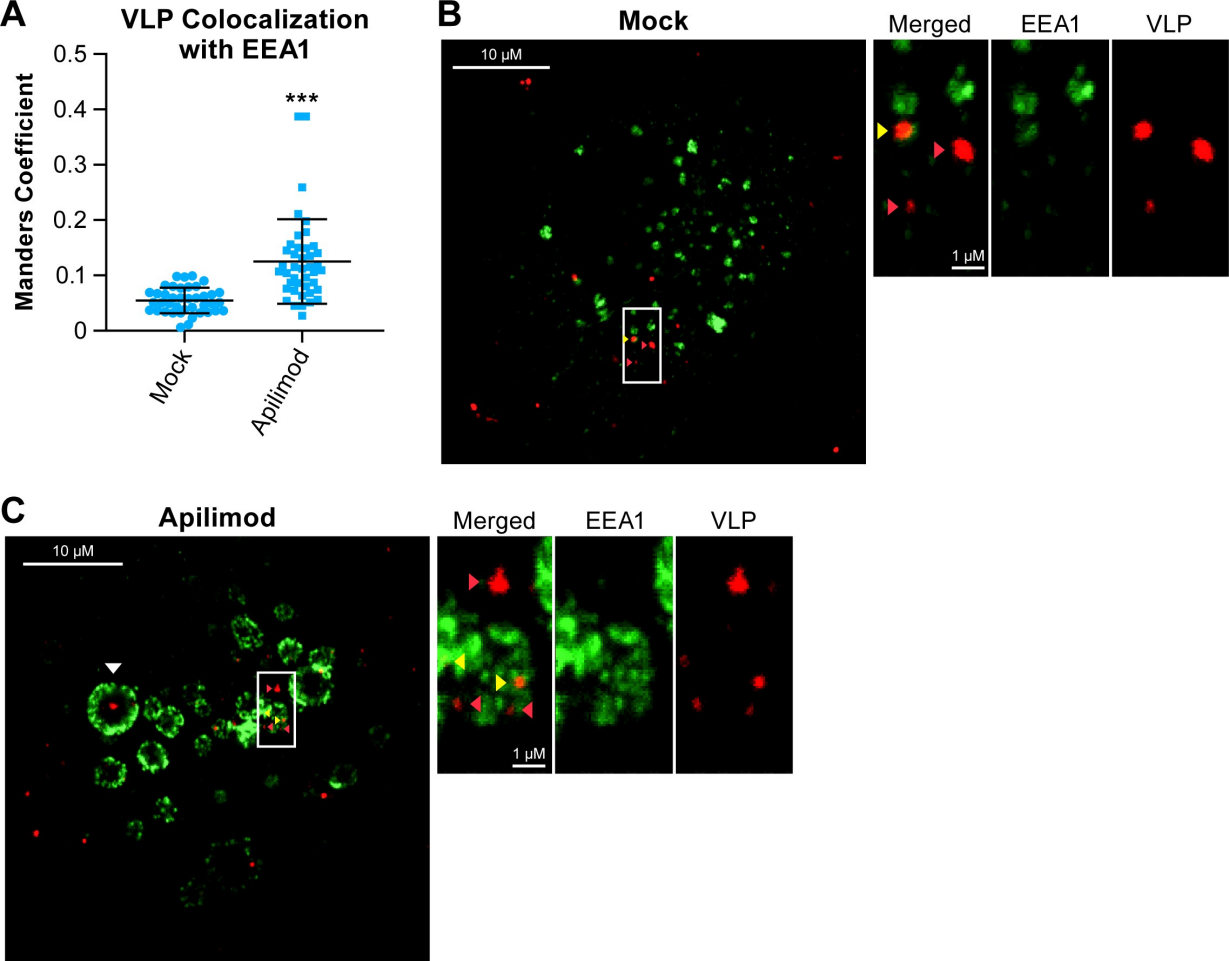
Apilimod causes accumulation of EBOV-GP VLPs in early endosomes. **(**A-C) BSC-1 cells were pretreated with DMSO (Mock) or 0.2 μM apilimod for 1 h at 37°C. Samples were analyzed for VLP colocalization with EEA1 at t = 90 min post initiation of VLP internalization. (A) Average colocalization coefficients (± SD) from 2 experiments (n = 40 and 42 fields for mock- and apilimod-treated cells, respectively). Each data point (blue dot, triangle or square) represents the Manders colocalization coefficient for 1 image field. (B and C) Representative micrographs of cells treated with (B) DMSO (Mock) or (C) 0.2 μM apilimod. Yellow arrows indicate areas of VLP colocalization with EEA1, and red arrows indicate VLPs that have not colocalized with EEA1. The white arrow (C, left panel) indicates a VLP that is within an enlarged EEA1^+^ endosome. Note that this VLP would not score as colocalized with EEA1. Hence the value for VLP colocalization with EEA1 in apilimod-treated cells is likely an underestimate. Scale bars, 10 μm (primary images) and 1μm (insets). ***p<0.001.

Concomitant with decreased trafficking of EBOV GP VLPs to NPC1^+^ endolysosomes, apilimod caused EBOV VLPs to accumulate in EEA1^+^ endosomes (Fig 6). In apilimod-treated cells, EEA1^+^ endosomes appeared larger than those in mock-treated cells, consistent with previous reports showing enlarged endosomes in cells genetically deficient for PIKfyve or treated with PIKfyve inhibitors [44,45,50,53].

**Fig 7:**
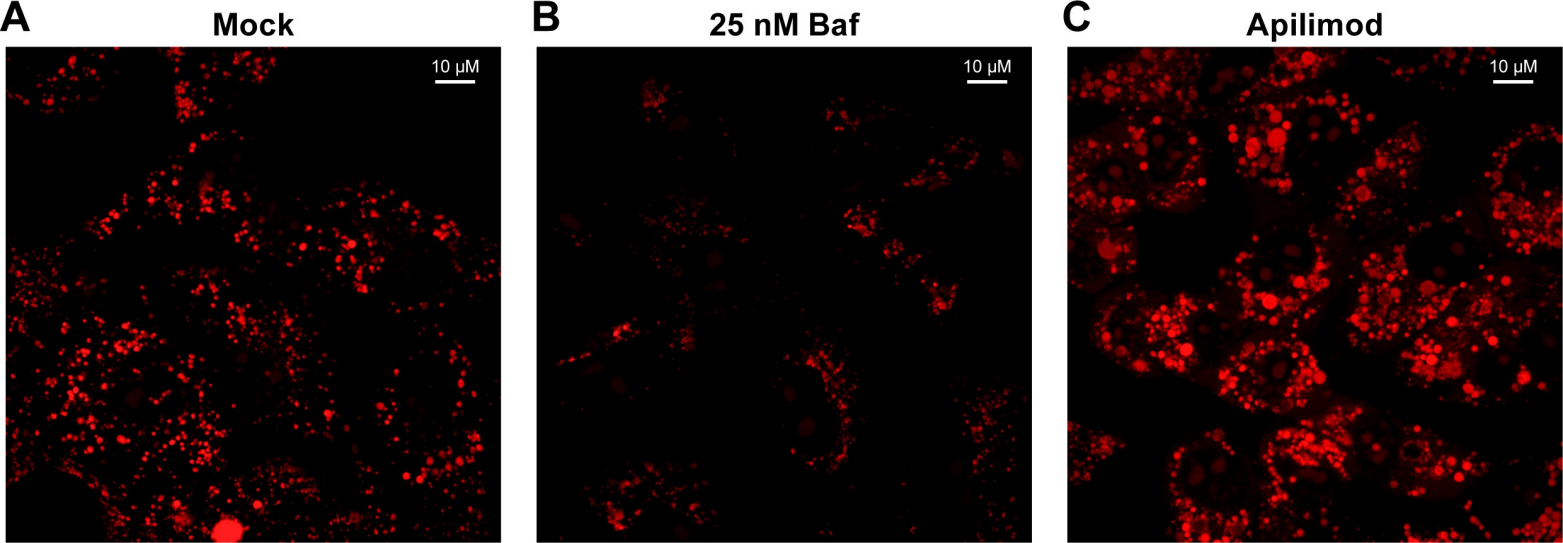
Apilimod has no effect on endosome acidification. BSC-1 cells grown overnight in 35 mm glass bottom dishes (MatTek) were treated for 3 h at 37°C with (A) DMSO (mock), (B) 25 nM bafilomycin (Baf), or (C) 0.2 μM apilimod. Acridine Orange (6.6 μg/mL) was added, and the cells were further incubated at 37°C for 20 min. The cells were then washed and imaged. Scale bars, 10 μm. Images are representative of all observed fields (30 for Baf and 35 for mock- and apilimod-treated cells, respectively).

**Fig 8:**
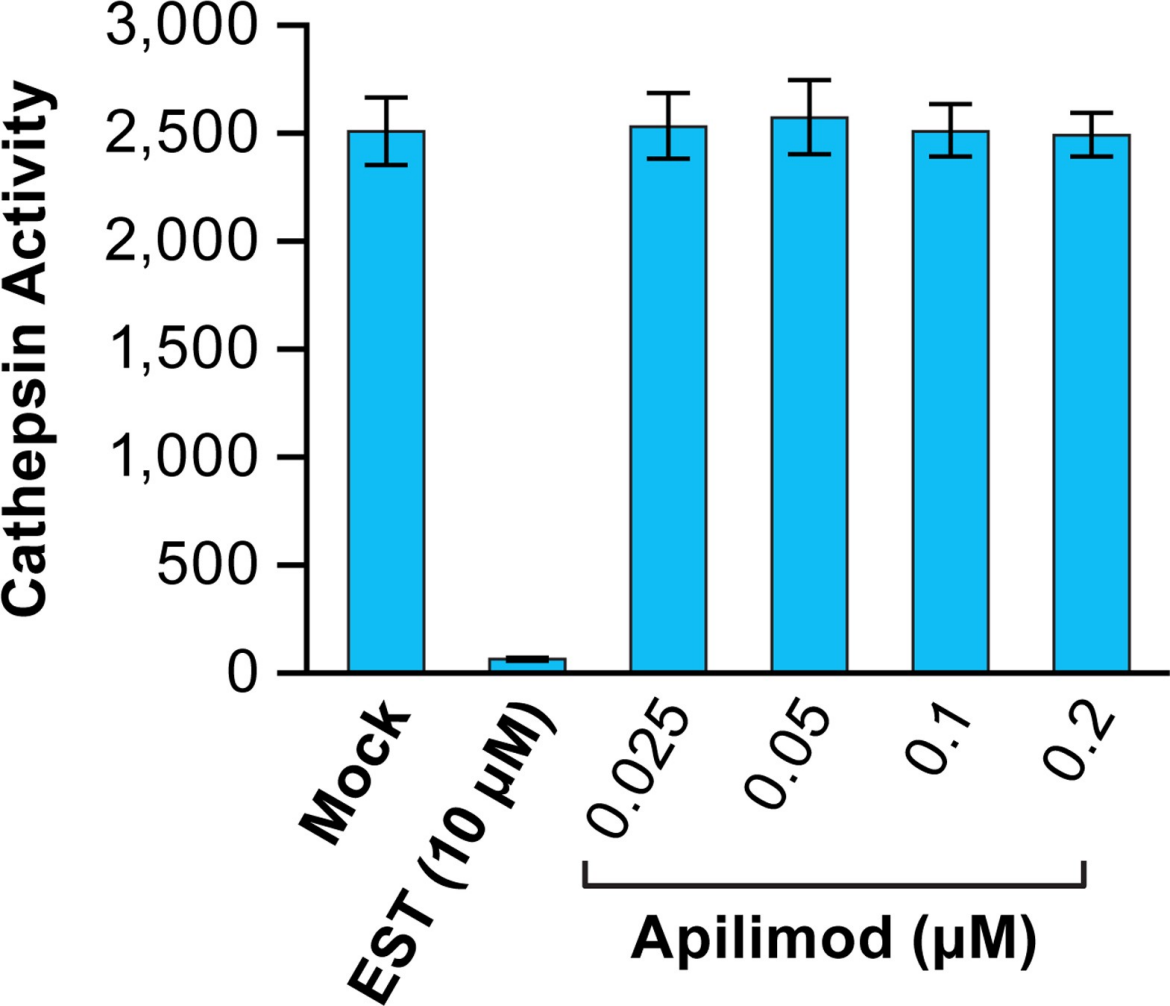
Apilimod has no effect on cathepsin B+L activity. Confluent dishes of BSC-1 cells were treated with the indicated concentration of apilimod, DMSO (mock), or 10 μM EST for 1 h at 37°C. The cells were then washed, lysed, and the pH adjusted to 5.0 in reaction buffer. Total cathepsin B+L activity was then assayed using the substrate Z-Phe-Arg-7-amido-4-methylcoumarin. Values represent average fluorescence units ± SD of triplicate samples.

**Fig 9:**
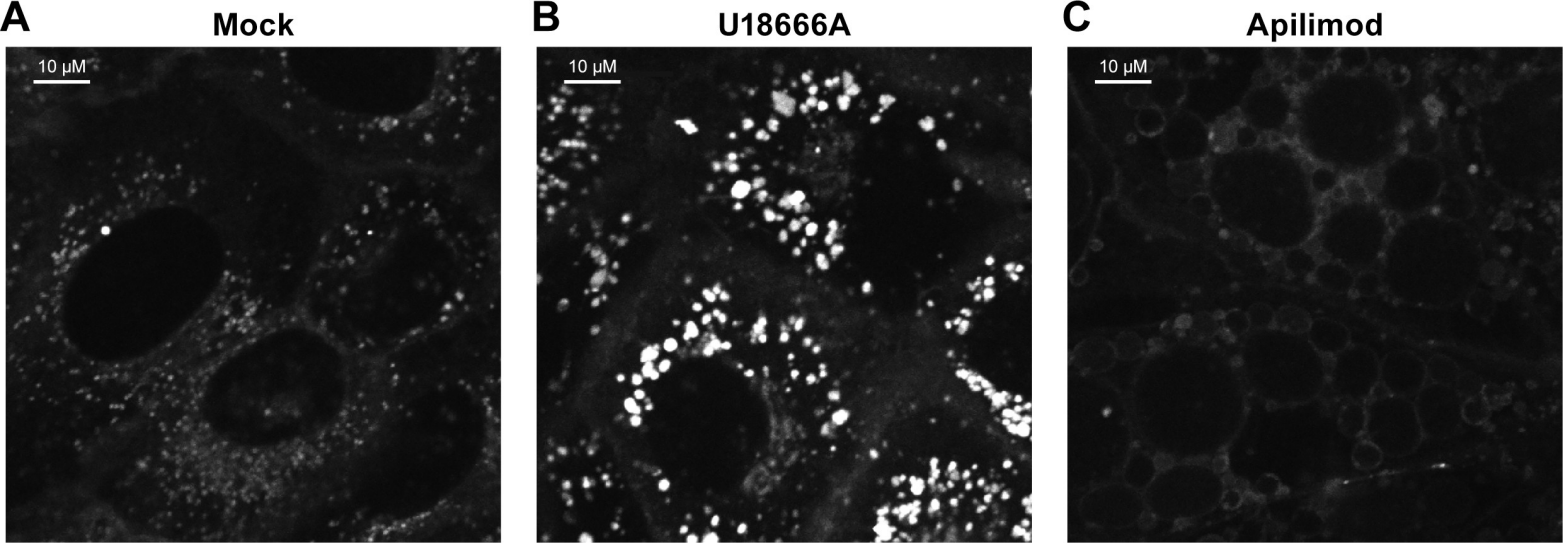
Apilimod does not induce cholesterol accumulation. BSC-1 cells were grown overnight on 35 mm glass bottom dishes (MatTek). The medium on the cells was replaced with serum-free medium containing 0.05 μM TopFluor Cholesterol (Avanti) plus (A) DMSO (mock), (B) 5 μM U18666A, or (C) 0.2 μM apilimod. The cells were incubated for 18 h at 37°C, washed once with PBS, and imaged in cell imaging medium. Images are representative of all observed fields (25 per treatment).

The findings presented in Figs 5 and 6 indicate that the primary mechanism by which apilimod blocks EBOV entry and infection (Figs 1–4) is by blocking virus transport from early (EEA1^+^) endosomes to their site of fusion in NPC1^+^ endolysosomes. To further test this model, we asked whether apilimod affects other attributes of the endosomal pathway needed for EBOV entry, either endosome acidification or the activity of cathepsin B and L [42,43]. At a concentration that strongly blocked EBOV entry and infection, apilimod had no detectable effect on endosome acidification (Fig 7). Bafilomycin, an inhibitor of EBOV infection that blocks endosome acidification, was used as a positive control. Apilimod also had no apparent direct effect on the activity of cathepsin B and L (Fig 8), in contrast to EST, a known inhibitor of cathepsin B, H, and L. Several cationic amphiphilic drugs such as U18666A that block EBOV entry and infection [20–22,24,25,68] induce cholesterol accumulation in endolysosomes [20,24]. In contrast, apilimod did not cause a detectable increase in cholesterol levels in endolysosomes (Fig 9). Hence apilimod appears to block filoviral entry and infection by inhibiting virus particle trafficking to NPC1^+^ endolysosomes, the portal for entry of the EBOV genome into the host cell cytoplasm [39–41].

## Discussion

As tragically demonstrated by the recent epidemic of EVD in Western Africa (2013–2016), a pressing need remains to develop therapeutics to treat patients infected with filoviruses [1]. While novel monoclonal antibody and small molecule therapeutics are in the pipeline [3–19], a parallel approach is to consider repositioning an approved drug or a drug that has proven safe in phase 2 clinical trials to treat EVD. In this study we tested the potential utility of apilimod as an anti-filoviral agent. We evaluated this drug for three reasons: (a) apilimod directly targets PIKfyve [54], a known EBOV entry factor [37]; (b) apilimod emerged from a blinded screen of 35 drugs targeting cell signaling pathways (S1 Fig and S1 Table); and (c) apilimod was reported to be well tolerated in humans in several phase 2 clinical trials [56–59]. After demonstrating that apilimod has activity against both EBOV and MARV in several cell types, notably in human macrophages (Figs 1 and 2, Table 1), we demonstrated that, by working through PIKfyve, its primary mode of action is to block trafficking of EBOV particles to NPC1^+^ endolysosomes (Figs 3–9), the site of EBOV fusion and entry [39–41].

### What is PIKfyve and how might it participate in EBOV entry?

EBOV journeys deep into the cellular endosomal system, entering the cytoplasm through endolysosomes that are positive for NPC1 and two-pore channel 2 (TPC2) [39]. In addition to NPC1, its intracellular receptor [14,37,38], EBOV requires multiple factors involved in endosome and lysosome biogenesis and maturation for entry [37]. One of the latter factors is PIKfyve [37], which phosphorylates PI3P to generate PI(3,5)P_2._ Here we have shown that apilimod, which binds to PIKfyve [54], blocks EBOV entry and infection in a PIKfyve-dependent manner.

The inhibitory effect of apilimod on EBOV entry is likely due to a defect in the maturation of endolysosomes, as extensive evidence indicates the importance of PIKfyve and PI(3,5)P_2_ in this process [44–53]. Although the exact mechanism by which PIKfyve and PI(3,5)P_2_ orchestrate endosome maturation is not known, several mechanisms have been postulated. Considered in these mechanisms are the observations that two Ca^++^ channels found in (endo)lysosomes—transient receptor potential cation channel, mucolipin 1 (TRPML1) [50,69] and TPC2 [70–72]—are downstream effectors of PIKfyve and PI(3,5)P_2_. Through its action on TRPML1, PIKfyve has been shown to regulate the fission and consequent remodeling and maturation that reduces the size of macropinosomes containing endocytosed material from the cell surface and exterior [50]. In addition, the TPC2 channel has been reported to be activated by PI(3,5)P_2_ [71,72]. Intriguingly, both macropinocytosis [35,73,74] and TPC2 [75] are involved in EBOV entry and infection. Although we do not yet know all of the endolysosomal factors needed to trigger EBOV GP for fusion [36,39,43,76], it appears clear that proper endosomal maturation is required. These findings are consistent with the mounting evidence for a role of PIKfyve in EBOV entry and our observation that the PIKfyve inhibitor, apilimod, blocks transport of EBOV particles to NPC1^+^ endolysosomes. It is therefore likely that interconnected effects of apilimod on PIKfyve [37,54], TPC2 [75], and endolysosome maturation culminate in its blockade of EBOV entry and infection.

### Potential utility of apilimod as an anti-filoviral agent

Several approved drugs that function as EBOV entry inhibitors (e.g., clomiphene, toremifene, and sertraline) block EBOV entry into the cytoplasm *after* EBOV particles have been delivered to NPC1^+^ endolysosomes [20,21,24,25]. Hence they likely interfere with some aspect of the virus-endolysosome membrane fusion process, *per se*. Other approved drugs, including chloroquine, niclosamide, atovaquone, amodiaquine and quinacrine [21–23], block endosomal acidification. Hence these drugs likely interfere with the processing of EBOV GP by acid-optimal endosomal cathepsins [42,43] and/or low pH-induced conformational changes required for fusion activity of cleaved GP [77,78].

In contrast to these mechanisms, our findings indicate that apilimod blocks EBOV entry by blocking particle delivery into NPC1^+^ endolysosomes. The only other approved drugs that we know of with anti-EBOV activity that are expected to have this mode of action are microtubule-disrupting agents, including colchicine, nocodazole, vinblastine, and vinorelbine [21–23]. Hence the mode of action of apilimod as an anti-filoviral agent is novel. Rather than blocking EBOV trafficking to NPC1^+^ endosomes by interfering with microtubules, apilimod blocks EBOV trafficking by inhibiting PIKfyve.

Our findings indicate that apilimod has similar anti-viral activity against EBOV and MARV, consistent with the need for NPC1 in endolysosomes for the entry of these and other filoviruses [37,79,80]. We therefore consider it likely that apilimod, a host-directed small molecule, will have broad or even pan-filoviral activity. Furthermore, since many other viruses, so-called late penetrating viruses [36,81], traffic beyond early endosomes for entry, it is possible that apilimod will block entry and infection by members of other virus families.

Apilimod is an investigational drug. Although it has been tested in phase 2 clinical trials for the treatment of Crohn’s disease, psoriasis, and rheumatoid arthritis, the drug has not yet been approved for any indication. Nonetheless apilimod was well-tolerated in humans in the reported phase 2 trials [56–59]. We found that intraperitoneal delivery of 10 mg/kg of apilimod to mice resulted in a C_max_ of 2.53 μM. This is well above the IC_50_ for apilimod inhibition of EBOV infection in the three cell lines tested, ~250 times greater than the IC_50_ in hMDMs (10 nM), initial major targets of filoviral infections [60,61]. We therefore consider it plausible that apilimod be used in the treatment of EVD. And, while apilimod may not function as a single agent, it may perform well as a component in an anti-filoviral small molecule cocktail. In summary, we introduce apilimod, a small molecule PIKfyve inhibitor that has proven safe in phase 2 clinical trials, as a potential anti-filoviral agent.

## Supporting information

S1 Fig Screen of 35 compounds using a reporter-expressing recombinant Ebola virus. Vero E6 cells were pretreated for 2 h with compounds at the indicated concentrations. Cells were then infected with 1000 TCID50 of a reporter-expressing Ebola virus in presence of the compounds, and 2 d later reporter activity (here shown on a log_10_ scale) was measured. In parallel, cell viability was determined in drug-treated but non-infected cells using a commercial cell viability assay, with viability in untreated cells set to 100%. As positive control, ribavirin was used at concentrations of 1 mg/ml, 100 μg/ml, and 10 μg/ml, and DMSO served as negative control. Mock indicates non-infected, untreated cells. Mean and standard deviation of 3 biological replicates are shown.
(TIF)

S1 Table Compounds blindly screened in the rgEBOV-luc2 infection assay (S1 Fig). Indicated are the screen code, NCGC number, name, and attributed mechanism of action for each compound. During the screen, apilimod (NCGC00263093-01) was coded D03; it is identified as apilimod in S1 Fig.
(DOCX)
