# Supplementary figures and images for: The phosphatidylinositol-3-phosphate 5-kinase inhibitor apilimod blocks filoviral entry and infection
Source: PLoS Negl Trop Dis. 2017 Apr 12;11(4):e0005540. doi: 10.1371/journal.pntd.0005540 (PMC5402990; doi:10.1371/journal.pntd.0005540)

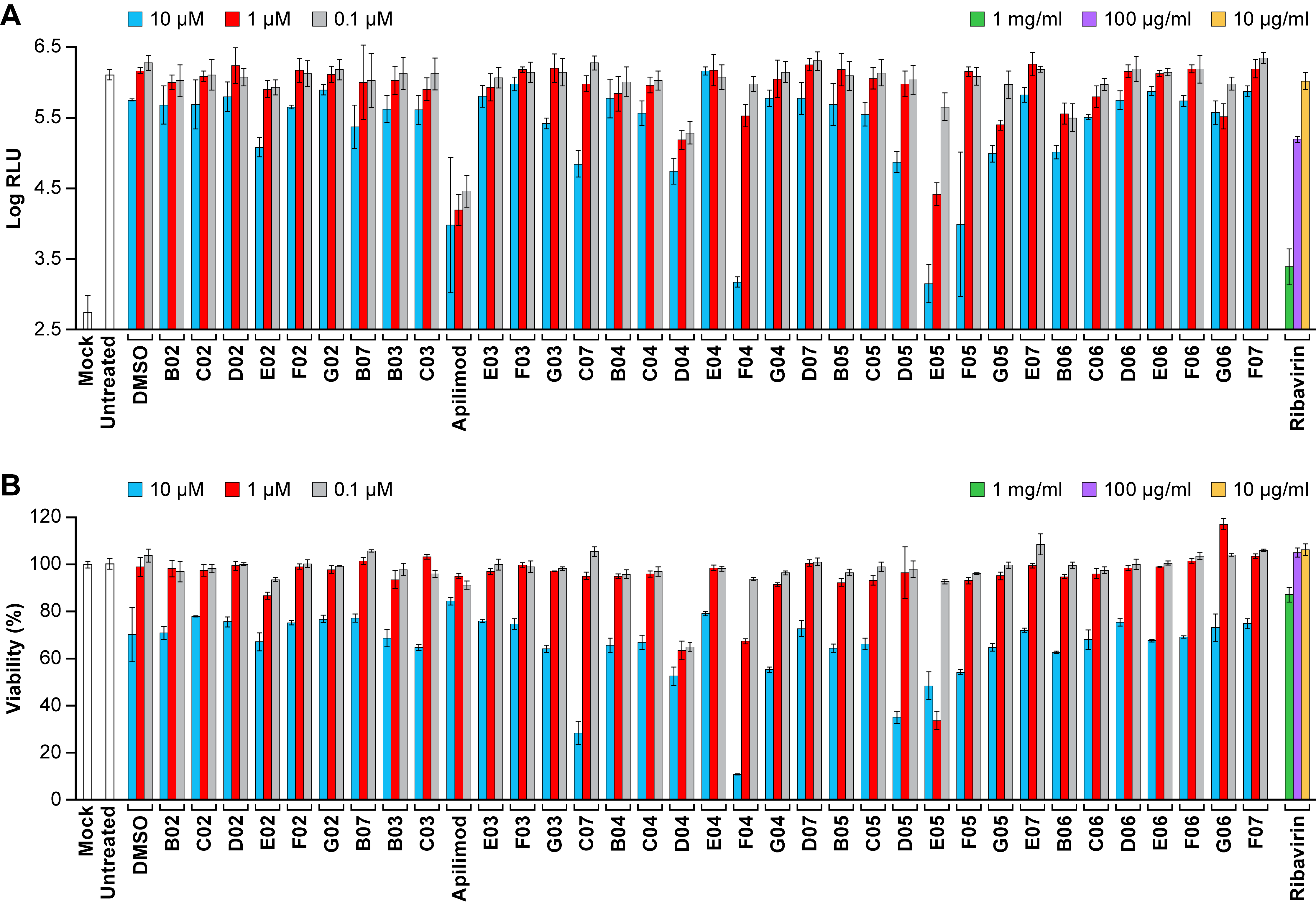

Supplement: S1 Fig — Vero E6 cells were pretreated for 2 h with compounds at the indicated concentrations. Cells were then infected with 1000 TCID50 of a reporter-expressing Ebola virus in presence of the compounds, and 2 d later reporter activity (here shown on a log10 scale) was measured. In parallel, cell viability was determined in drug-treated but non-infected cells using a commercial cell viability assay, with viability in untreated cells set to 100%. As positive control, ribavirin was used at concentrations of 1 mg/ml, 100 μg/ml, and 10 μg/ml, and DMSO served as negative control. Mock indicates non-infected, untreated cells. Mean and standard deviation of 3 biological replicates are shown. (TIF) [file pntd.0005540.s001.tif]
