# Supplementary material for: The phosphatidylinositol-3-phosphate 5-kinase inhibitor apilimod blocks filoviral entry and infection
Source: PLoS Negl Trop Dis. 2017 Apr 12;11(4):e0005540. doi: 10.1371/journal.pntd.0005540 (PMC5402990; doi:10.1371/journal.pntd.0005540)
Supplement: S1 Table — Indicated are the screen code, NCGC number, name, and attributed mechanism of action for each compound. During the screen, apilimod (NCGC00263093-01) was coded D03; it is identified as apilimod in S1 Fig. (DOCX) [file pntd.0005540.s002.docx]

| **Code** | **Drug** | **Name** | **Mechanism of Action** |
| --- | --- | --- | --- |
| B02 | NCGC00229511-04 | Tofactinib | JAK inhibitor |
| C02 | NCGC00274171-12 | RUC-4 | Allbβ3 agonist |
| D02 | NCGC00347957-02 | UNC-669 | MBT domain antagonist |
| E02 | NCGC00250406-01 | Tipifarnib | Farnesyltransferase inhibitor |
| F02 | NCGC00346966-01 | XMD13-2 | Receptor-interacting serine/threonine protein kinase 1 inhibitor |
| G02 | NCGC00025297-04 | Gabexate mesylate | Serine protease inhibitor |
| B07 | NCGC00238451-04 | Olapanib | PARP inhibitor |
| B03 | NCGC00263141-01 | STF-083010 | IRE1α endonuclease activity inhibitor |
| C03 | NCGC00090762-03 | Rifabutin | Antibiotic; inhibitor of prokaryotic RNA polymerase |
| E03 | NCGC00164591-02 | **Artemether** | **antimalarial** |
| F03 | NCGC00165783-02 | Vadimezan | Tumor-vascular disrupting agent; VEGFR2 inhibitor |
| G03 | NCGC00263190-02 | KU 55933 | ATM inhibitor |
| C07 | NCGC00263621-02 | I-BET-762 | BET inhibitor |
| B04 | NCGC00022678-03 | Rifampicin | Antibiotic; inhibitor of prokaryotic RNA polymerase |
| C04 | NCGC00163485-03 | 17β-hydroxy Wortmannin | PI3K inhibitor |
| D04 | NCGC00162440-02 | Leptomycin B | CRM1/eportin 1 inhibitor |
| E04 | NCGC00165844-02 | NU-6027 | CDK1/2 inhibitor, ATR inhibitor |
| F04 | NCGC00344588-02 | I-BET151 | BDR2, BDR3, BDR4 inhibitor |
| G04 | NCGC00345784-01 | PF-3758309 | PAK1, PAK3 inhibitor |
| D07 | NCGC00344622-02 | SGC-0946 | DOT1L inhibitor |
| B05 | NCGC00346444-01 | VE 821 | ATR inhibitor |
| C05 | NCGC00346435-01 | Niraparib | PARP inhibitor |
| D05 | NCGC00094792-06 | Tioguanine | Amidophosphoribosyltransferase inhibitor; purine synthesis inhibitor |
| E05 | NCGC00025059-04 | Actinomycin D | DNA-directed RNA polymerase inhibitor |
| F05 | NCGC00346070-02 | GSK2606414 | PERK inhibitor |
| G05 | NCGC00345803-01 | AVN-944 | Inosine monophosphate dehydrogenase inhibitor |
| E07 | NCGC00344623-02 | UNC1215 | L3MBTL3 methyllysine (Kme) reader domain inhibitor |
| B06 | NCGC00250383-01 | Irestatin 9389 | IRE1α endonuclease activity inhibitor |
| C06 | NCGC00070736-04 | Imiquimod | TLR7 receptor antagonist |
| D06 | NCGC00346532-01 | Lomibuvir | RNA-directed RNA polymerase inhibitor |
| E06 | NCGC00346463-01 | Nesbuvir | RNA-directed RNA polymerase inhibitor |
| F06 | NCGC00244253-01 | Ruxolitinib | JAK1/2 inhibitor |
| G06 | NCGC00345794-01 | MLN4924 | NEDD8-Activating Enzyme (NAE) Inhibitors |
| F07 | NCGC00347943-02 | GSK J1 | H3K27 Histone Demethylases UTX and JMJD3 Inhibitor |

**Supporting Information. Table 1.** Compounds blindly screened in the rgEBOV-luc2 infection assay (Supporting Information, Fig. 1). Indicated are the code, NCGC number, name, and attributed mechanism of action for each compound. During the screen, apilimod (NCGC00263093-01) was coded D03, it is identified as apilimod in Supporting Information, Fig. 1.
